# Restrained Stress Development in Hardening Mortar Internally Cured with Superabsorbent Polymers under Autogenous and Drying Conditions

**DOI:** 10.3390/polym13060979

**Published:** 2021-03-23

**Authors:** Jung Heum Yeon

**Affiliations:** Department of Civil and Environmental Engineering, Gachon University, Seongnam 13120, Korea; jyeon@gachon.ac.kr; Tel.: +82-31-750-5498

**Keywords:** superabsorbent polymers, internal relative humidity, autogenous shrinkage, drying shrinkage, restrained stress, cracking, strength, set time

## Abstract

This study reports the results of a series of experiments, particularly paying attention to the early-age behavior and response of hardening mortars incorporating different types and contents of superabsorbent polymer (SAP) under autogenous (sealed) and drying shrinkage (unsealed) conditions. To achieve this primary aim, the effects of SAP type (i.e., cross-linking density and grain size) and content on the internal relative humidity (IRH) changes and corresponding free shrinkage behavior, restrained stress development, and cracking potential of the mortar were extensively measured and analyzed, along with their strength and set time properties. The results of this study have shown that the internal curing (IC) via SAP effectively counteracted the early-age residual stress build-up due to autogenous shrinkage, as many other former studies described. No or little tensile residual stresses due to autogenous shrinkage took place when more than 0.4% SAP was added, regardless of the SAP type. However, it should be mentioned that the addition of SAP, irrespective of its content and type, hardly improved the shrinkage cracking resistance of the mortar when directly exposed to drying environment at early ages.

## 1. Introduction

Most of the modern concrete mixtures used in the construction industry are engineered to have enhanced mechanical properties, high fracture toughness (energy absorption capacity), prolonged fatigue life, and improved durability to reach the current sustainable development goals. For instance, ultra-high-performance concrete (UHPC) mixtures are required to meet specific performance criteria such as 28-day compressive strength of >150 MPa and 28-day tensile strength > 8 MPa, along with strain-hardening and multiple cracking behavior [1]. A fundamental approach to produce such high-strength and durability concrete mixtures is to adopt a low water-to-binder ratio (w/b), generally less than 0.30. However, one of the most common issues associated with the use of low w/b is the propensity to allow a large amount of early-age shrinkage caused by self-desiccation, which is the phenomenon that occurs when unhydrated cement in the matrix continues to chemically bind with water trapped in the pores for delayed hydration [2,3]. Such volume reduction in the hydration products eventually leads concrete to crack if it is resisted by internal and/or external restraints [4]. Accordingly, it is crucial to adequately control the shrinkage cracking at early ages for broad applications of low-w/b concrete mixtures in the construction practice.

A number of research attempts have been made to reduce the restrained shrinkage development and shrinkage cracking problems in early-age concrete. Some existing studies [5,6] reported that the use of expansive cement results in restrained volume expansion of concrete by forming a large amount of ettringite, which in turn compensates the subsequent shrinkage. Another investigation [7] described that a shrinkage-reducing agent reduces the surface tension of pore water, and thus can offset the shrinkage cracking potential of concrete. Swayze [8] proposed a method to tackle the autogenous shrinkage development by taking advantage of thermal dilation and improved mechanical properties through heat of hydration of cement. Another study [9] described that the use of high-modulus aggregate effectively resisted to the shrinkage development in concrete. A latest study by Szostak and Golewski [10] demonstrated that the use of a nanoadmixture containing nanometric C–S–H seeds can reduce drying shrinkage due to postponement of the beginning of matrix shrinkage via earlier intensification of exothermic reactions. It was also reported that external moisture supply via water ponding and/or fog misting during early hydration stages can also be a good option to mitigate the shrinkage cracking potential, but the reduced capillary porosity and early depercolation in low-w/b mixtures may result in limited curing effectiveness [11]. Recently, internal curing (IC) via highly-absorptive materials has been receiving an enormous attention and interest of researchers and practitioners as an active measure to counteract the autogenous shrinkage occurring in low w/b mixtures. On top of the shrinkage limiting capability, the IC reportedly allows cement to promote its degree of hydration, and thus helps concrete to better develop the potential properties, especially in terms of long-term strength and durability [12]. Many studies have made an attempt to use lightweight aggregate (LWA) as an IC agent due to its high absorptivity. Castro et al. [13] investigated the absorption and desorption characteristics of fine LWA for applications to internally cured concrete mixtures. Lura et al. [14] examined the efficiency of IC with LWA produced from biomass-derived waste via neutron tomography, internal relative humidity (IRH) and autogenous deformation measurements. Balapour et al. [15] evaluated the potential use of LWA produced from bottom coal ash for IC of concrete. Zhang and Poon [16] reported the IC effect of high-volume furnace bottom ash incorporation on LWA.

Lately, studies on superabsorbent polymers (SAP) for IC of high-performance cement-based materials have been actively performed. SAP is a kind of new class functional material that can hold a substantial amount of water (up to 500 times relative to its own mass) through hydrogen bonding, and thus is used as a novel IC agent to control the early-age shrinkage in high-performance concrete mixtures. Since Jensen and Hansen pioneered research on the IC of concrete via SAP back in 2001 [17,18], extensive studies have been followed up to see how the SAP addition affects the various properties including autogenous shrinkage [19,20,21], autogenous shrinkage-induced crack control [22,23], mechanical properties [22,24,25,26], creep [27], rheology [28,29,30], self-sealing and healing capabilities [30,31,32,33,34,35], coefficient of thermal expansion [36,37], freeze-thaw durability [38,39]. In addition, fundamentals of the absorption and desorption characteristics were studied [19,40,41].

## 2. Research Significance

Although massive studies were recently conducted for SAP and its potential uses, most of the works mainly focused on its autogenous shrinkage reducing capability. Little research effort has been made to assess how the mixture internally cured with SAP behaves when subjected to early exposure to drying environment. In this study, the early-age behavior and response of cement mortars incorporating different types and contents of SAP are investigated, especially focusing on the influence of early external drying. The experimental programs consist of IRH and corresponding free shrinkage measurements, calculation of residual stress development under a restrained shrinkage condition, and monitoring of shrinkage cracking potential. Moreover, the compressive and flexural strengths as well as initial and final set times are measured for systemic analysis of the obtained results.

## 3. Materials and Methods

### 3.1. Materials

Type I Portland cement specified in ASTM C150 [42] was used. The chemical composition and physical properties of the cement are shown in Table 1. The phase compositions of the cement calculated based on the Bogue equations were 60.65% C3S, 10.72% C2S, 9.22% C3A, and 8.82% C4AF. As fine aggregate, standard sand conforming to KS F 2406 was used. The fine aggregate used had a median diameter (D50) of 533 μm, a specific gravity of 2.65, a fineness modulus of 2.87, an absorption capacity of 1.02%, and a SiO_2_ content of 98.4%. A recommended dosage (5 mL/cwt) of lignosulfonate-based water reducer was added.

**Figure 1:**
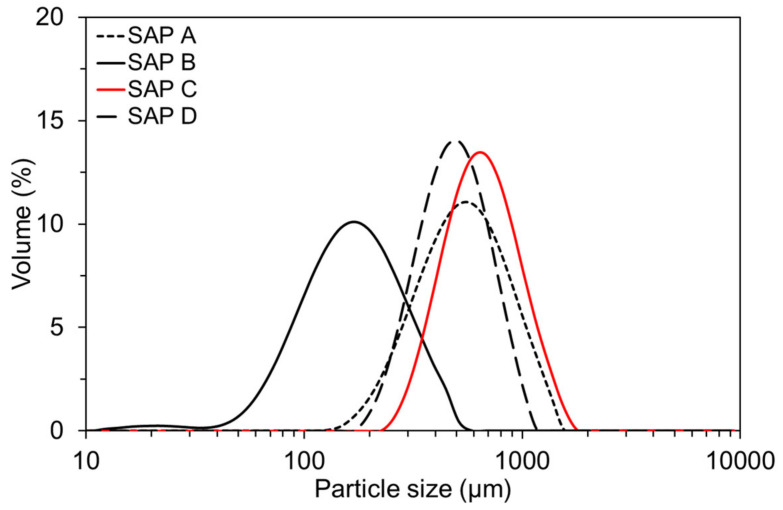
Particle size distributions of SAPs used [41].

**Figure 2:**
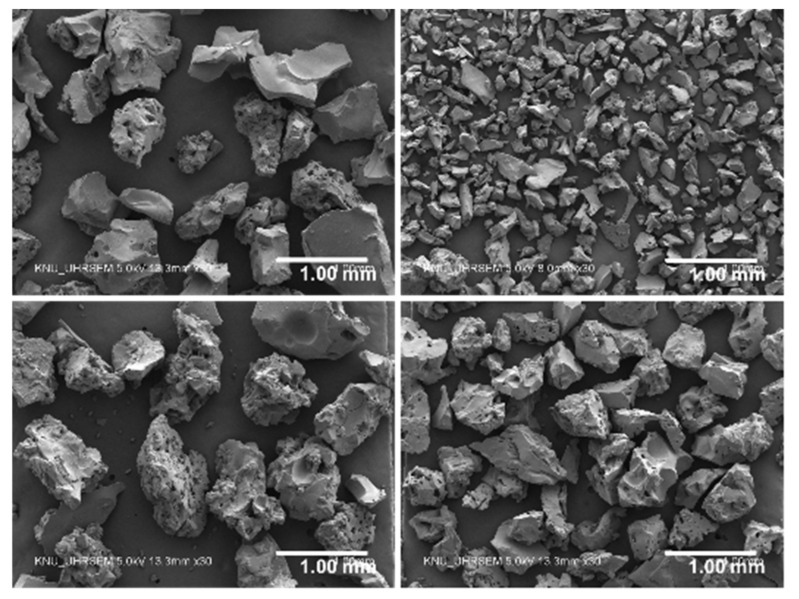
Scanning electron microscope images of SAP A, SAP B, SAP C, and SAP D for 5.0 kV 8.0 mm × 30 (clockwise from top left, respectively) [41].

As a water absorbing agent for IC, four types of sodium polyacrylate-based SAP with different dry grain sizes and cross-linking densities were used. The general information of the SAP is given in Table 2. The SAP was made of >92% sodium polyacrylate and <8% water, which were polymerized in bulk first, followed by pulverized into irregular-shape powders. The particle size distributions (Hydro 2000S; Malvern Instruments, Malvern, UK) and the scanning electron microscopy (SEM) images (Hitachi S-4800; Hitachi, Chiyoda, Japan) of the SAP are presented in Figure 1 and Figure 2, respectively. The mean dry particle diameter was 535.01 µm, 165.84 µm, 644.84 µm, and 482.37 µm for SAP A, SAP B, SAP C, and SAP D, respectively. The chemical and physical characteristics of the SAP are shown in Table 3.

Table 4 shows the mixture proportions of cement mortar used in this study. The effective water-to-cement ratio (w/c) (the w/c calculated based on the free water portion only) and fine aggregate-to-cement ratio (a/c) was consistent with 0.3 and 2.75, respectively. The dosage of SAP was 0.2%, 0.4%, and 0.6% by mass of cement, which was determined based on the preliminary investigations on the absorptivity and mechanical properties. The total water content (free water + IC water) was adjusted per the absorption capacity of each SAP measured in a previous study [41]. The absorption capacity under actual mixing condition was 12.70 g/g for SAP A, 8.75 g/g for SAP B, 4.82 g/g for SAP C, and 10.99 g/g for SAP D. Mixing of the mortar mixtures was done using a 5-L Hobart-type mixer following the standard procedure. SAP was dry-mixed with cement and sand before mixing water was added.

### 3.2. Methods

#### 3.2.1. Set Times

**Figure 3:**
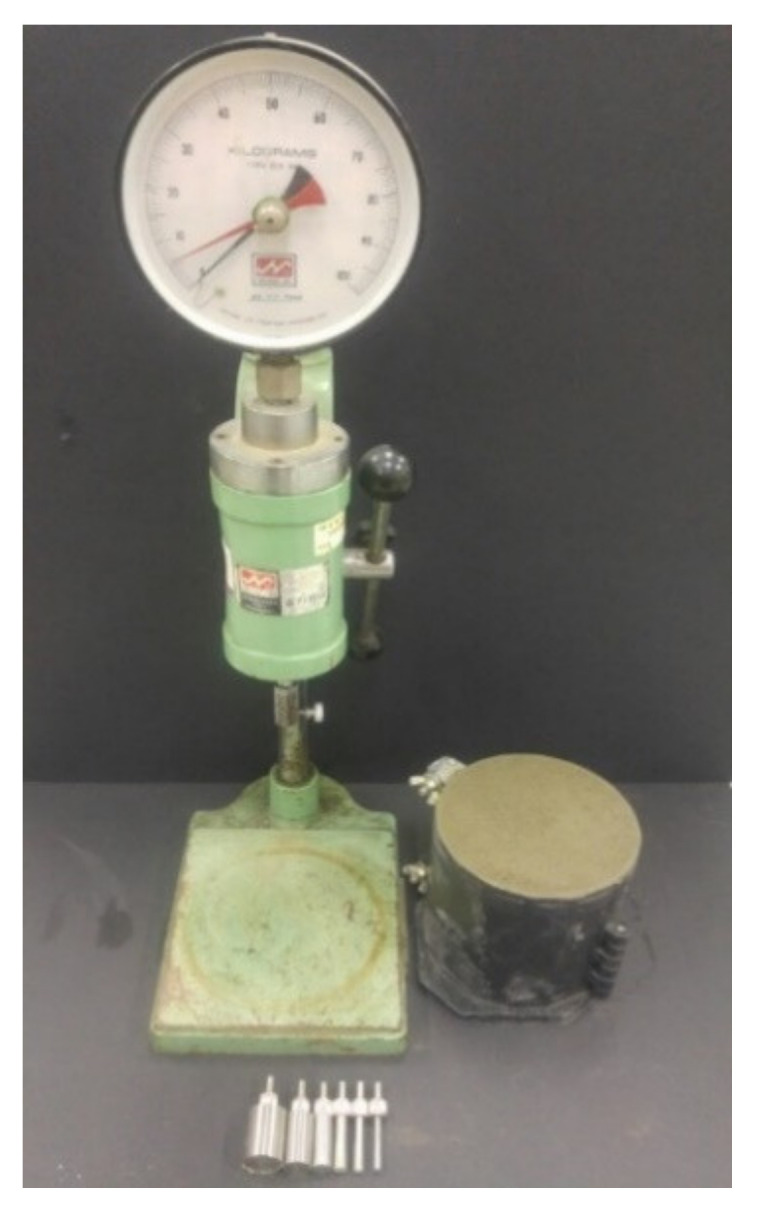
Test setup for set time measurement.

The initial and final set times of cement mortar were estimated based on ASTM C403-16 [43] (Standard Test Method for Time of Setting of Concrete Mixtures by Penetration Resistance) in order to determine the zeroing time where a restrained stress starts to develop in hardening mortar. A spring-reaction type loading device and cylindrical mold with dimensions of *Φ*150 × 150 mm were used to measure the changes in penetration resistance over time as shown in Figure 3. The initial and final set times were determined by interpolating the obtained data points when the penetration resistance reached 3.5 MPa and 27.6 MPa, respectively. An exponential function was used to reliably fit the data points as per the ASTM standard.

#### 3.2.2. Strengths

The flexural strength was measured conforming to ASTM C348 [44] (standard test method for flexural strength of hydraulic-cement mortars). Three replicate prismatic specimens with dimensions of 40 mm × 40 mm × 160 mm were fabricated for each experimental variable, and they were cured at 23 ± 0.5 °C and 95% RH for 24 h. Subsequently, the specimens were demolded and stored in dual air-tight plastic bags to retain moisture until the specified test ages are reached, i.e., 1, 3, 7 and 28 days. The flexural strength was measured by center point loading with a constant load rate of 50 N/s using a 200-kN universal testing machine (UTM).

The compressive strength was measured as per ASTM C349 [45] (standard method for compressive strength of hydraulic-cement mortars using portions of prisms broken in flexure) using the specimens broken in two parts upon completion of the flexural test. A total of six specimens were tested for each variable with a load rate of 2400 N/s. The load bearing area was 40 mm × 40 mm.

#### 3.2.3. Internal Relative Humidity and Autogenous Shrinkage

The bulk linear autogenous shrinkage of mortar containing different types and contents of SAP was measured. Simultaneously, the internal relative humidity (IRH) was measured as a direct measure of moisture-induced volume changes. A vibrating wire strain gage (VWSG) (Model 1240; ACE Instrument Co., Ltd., Gunpo, Korea) connected to a multi-purpose data logger (CR1000; Campbell Scientific, Inc., Logan, UT, USA) was longitudinally situated at the geometric center of the cross-section to measure the sectional average shrinkage strain in bulk. The VWSG had a capacity of 3000 με, a gage length of 153 mm, and a resolution of 0.5 με. Temperature corrections were made using a built-in thermistor to convert the measured strain to the true strain. A capacitance-type relative humidity sensor (HYT-939; IST AG, Ebnat-Kappel, Switzerland) was used for the IRH measurement. The sensor had an accuracy of ±1.8% RH and ±0.2 °C. A 16-channel data logger (MSHTDL-16) was used to log the IRH data at a specified interval. The IRH sensor was treated with a moisture-transmittable engineered fabric that allows water vapor exchange while blocking direct water pass.

**Figure 4:**
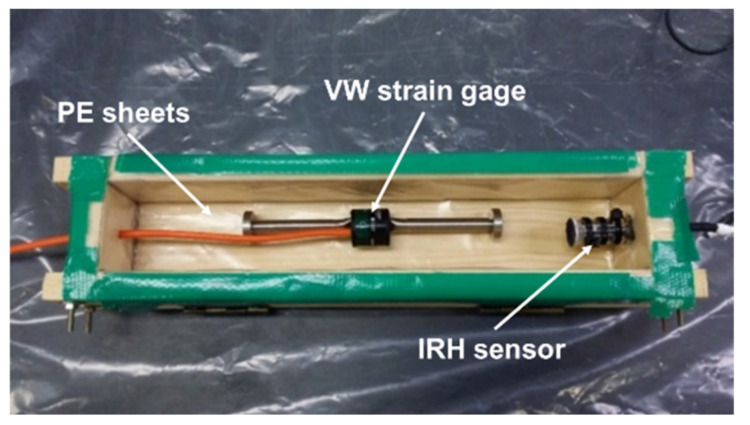
Test setup for autogenous shrinkage and internal relative humidity (IRH) measurements.

**Figure 5:**
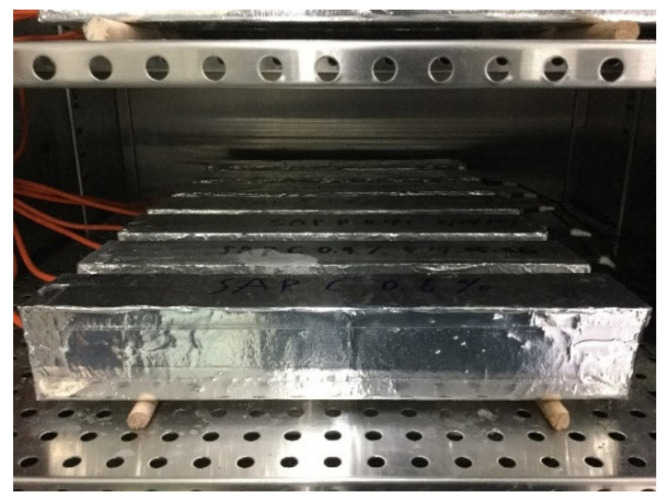
Autogenous shrinkage and IRH measurements in progress.

To fabricate the shrinkage specimen, fresh mortar was poured into a prismatic mold with inner dimensions of 50 mm × 50 mm × 300 mm in two layers. Each layer was vibrated for 30 sec by means of a vibrating table after manually compacting 25 times using a rigid rod. To minimize the friction between the specimen and mold during length changes, a double layer of polyethylene sheets was placed onto the inner surfaces of the mold. After 24 h, the specimen was demolded and then promptly sealed with adhesive-backed aluminum foil to prevent a moisture loss. Subsequently, all the specimens were stored in an environmental chamber maintaining 23 ± 0.5 °C while collecting the data using data loggers. Two wooden roller supports were placed underneath the specimen to allow the free deformation triggered by self-desiccation. Figure 4 and Figure 5 illustrate the test setup for autogenous shrinkage and IRH measurements and the specimens stored in the chamber, respectively.

#### 3.2.4. Restrained Ring Test

**Figure 6:**
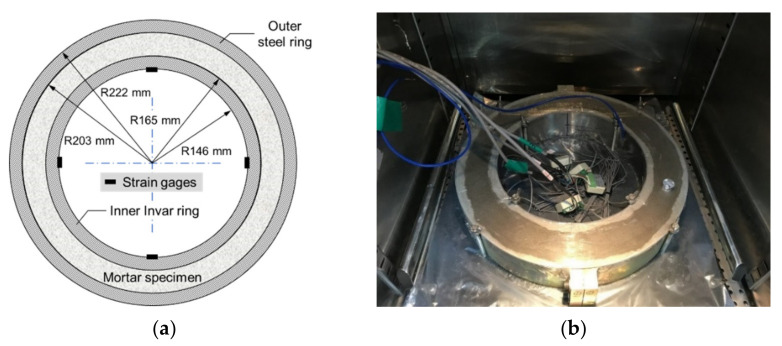
Restraining ring test apparatus: (**a**) geometry; (**b**) actual test setup.

**Figure 7:**
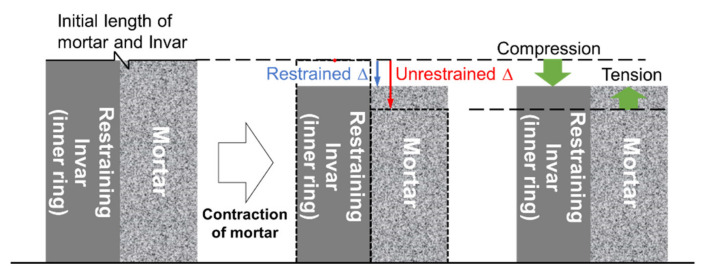
Conceptual schematic of operating principle of restrained ring [48].

A restrained ring test device modified on the basis of ASTM C1581 [46] (standard test method for determining age at cracking and induced tensile stress characteristics of mortar and concrete under restrained shrinkage) was used to quantify the restrained stress development in hardening mortar. The inner ring of this device was made with Invar 36 (FeNi36; also known as nickel-steel alloy) with a notably low coefficient of thermal expansion (about 1/10) instead of conventional structural steel (Grade B steel) because the thermal deformation of the inner ring can be kept negligible, thereby producing a stable degree of restraint during test. The nickel content and coefficient of thermal expansion of the Invar used was 35.9 wt % and 1.57 × 10^−6^ mm/mm/°C at 30–100 °C, respectively. The elastic modulus of the Invar was 141 GPa. Another piece of ring with a larger diameter made of the conventional structural steel was concentrically placed outside of the inner ring. Fresh mortar was cast between the inner and outer rings in two layers; each layer was manually compacted 25 times followed by mechanical vibration for 30 s. Figure 6a,b presents the geometry of the test apparatus and actual test setup, respectively. The thickness of the mortar specimen and restraining ring was 38 and 19 mm, respectively, which resulted in a degree of restraint of the restraining ring of approximately 72% [47] comparable to that of the standard ring as per ASTM C1581. The height of both specimen and inner/outer rings was 79.2 mm. Figure 7 illustrates how the restraint provided by the inner ring induces tensile stress in the surrounding mortar specimen upon shrinkage. Four steel strain gages (FLA-5-11; Tokyo Measuring Instruments Laboratory Co., Ltd., Tokyo, Japan) with a gage length of 5 mm and a resistance of 120 Ω were circumferentially attached on the inner surface of the inner ring at a 90° interval to measure the tangential elastic strain of the inner ring caused by the shrinkage of the specimen.

**Figure 8:**
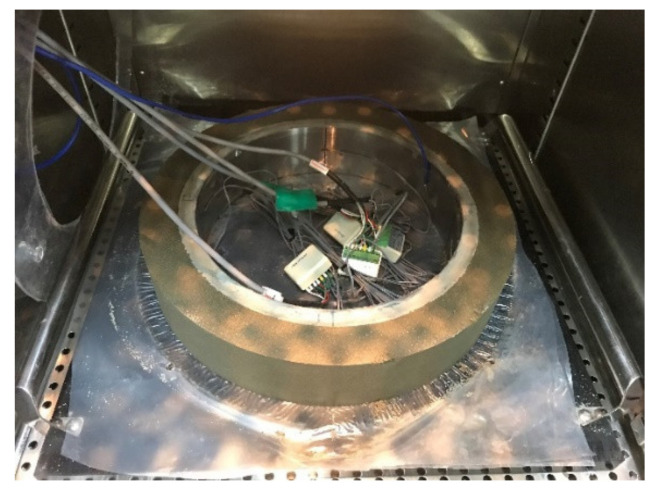
Measurement of restrained stress development due to external drying (right before attaching adhesive-backed aluminum foil on the top surface).

The restrained ring tests were performed in two different schemes. First, fresh mortar was cast in a mold and was completely sealed for moist curing at 23 ± 0.5 °C for 24 h. Subsequently, the specimen was allowed to dry through the outer circumference at 23 ± 0.5 °C and 60 ± 1% RH until it cracked to see how the early drying affects the restrained stress development and cracking potential of the mortar incorporating SAP. Second, the specimen was initially cured in a sealed condition (autogenous condition), and then was exposed to controlled drying environment (drying condition) in order to assess the restrained stress development and cracking risk due to autogenous shrinkage and delayed external drying, considering conventional formwork-removal operations in the field. The drying commenced 100 h after final set at 23 ± 0.5 °C and 60 ± 1% RH. Similarly, with the first experimental scheme, the specimen was dried through the outer circumference only whereas the top, bottom, and inner surfaces were tightly sealed with adhesive an aluminum foil sheet, reinforced-plastic bottom plate, and outer steel ring, respectively, as can be seen in Figure 8. Once the average strain was obtained from the four strain gages, the circumferential restrained stress in the ring specimen can be calculated as follows [44]:

(1) $$\sigma_{\theta }\left(R_{IC}\right)=-\varepsilon_{in}E_{invar}\left[\frac{R_{IC}^{2}-R_{II}^{2}}{2R_{IC}^{2}}\right]\left[\frac{R_{OC}^{2}+R_{IC}^{2}}{R_{OC}^{2}-R_{IC}^{2}}\right]$$

where $\sigma_{\theta }\left(R_{IC}\right)$ is the circumferential restrained stress in the specimen (MPa); $\varepsilon_{in}$ is the strain measured on the inner ring (µε); $E_{invar}$ is the elastic modulus of the Invar (MPa); $R_{IC}$ is the inner face radius of the specimen (mm); $R_{II}$ is the inner face radius of the inner restraining ring (mm); and $R_{OC}$ is the outer face radius of the specimen (mm).

## 4. Results and Discussion

### 4.1. Effects of SAP Type and Content on the Set Times

**Figure 9:**
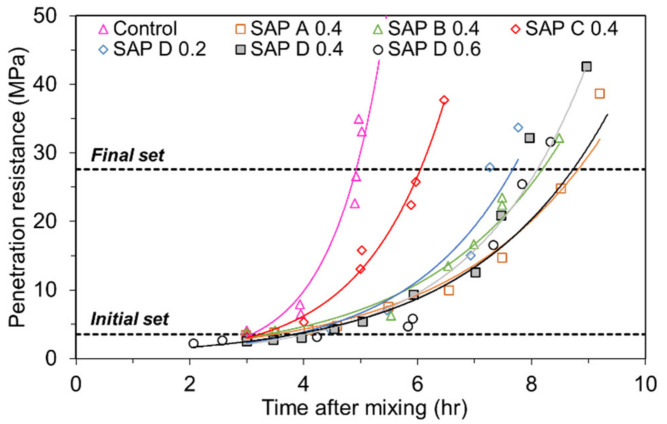
Results of penetration resistance test with exponential fitting curves.

**Figure 10:**
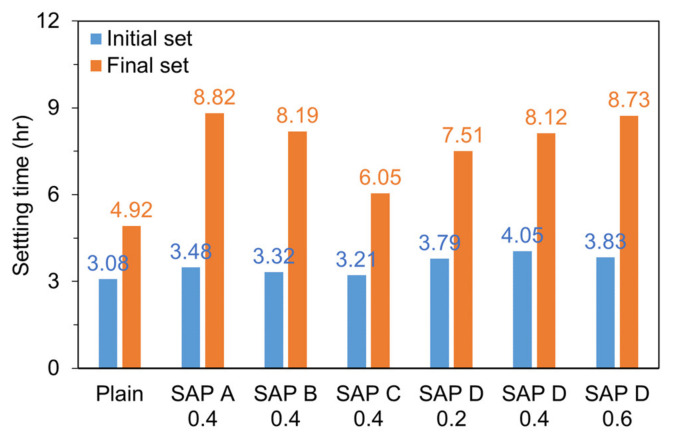
Initial and final set times for each mixture estimated based on fitting functions.

Figure 9 and Table 5 show the changes in penetration resistance with different types and contents of SAP and their fitting parameters for the exponential regression curves, respectively. Additionally, Figure 10 compares the initial and final set times for each mixture estimated based on the regression functions. Note that it took a shorter time for SAP C 0.4 to reach both the initial and final set than SAP A 0.4, indicating that the greater the cross-linking density, the shorter the set times when the grain size is similar with each other. This is presumably because as the cross-linking density increases, a higher level of retraction forces acts in the network structure, which empties the entrained water in SAP faster, and in turn raise the degree of hydration of cement [41,49]. Furthermore, it appears that the stronger gel strength of SAP C contributed to the faster stiffness gain of SAP C 0.4 than SAP A 0.4. When compared the results of SAP B 0.4 with those of SAP D 0.4, which has a similar cross-linking density but a different grain size, SAP B 0.4 showed a 18% shorter time than SAP D 0.4 to reach the initial set. This result was obtained because SAP B had a faster desorption rate than SAP D and thus promoted the degree of hydration of cement at very early ages, which well coincided with the results of the former study [41]. On the other hand, no meaningful difference was found in the final set time between SAP B 0.4 and SAP D 0.4. The results of the SAP D series (i.e., SAP D 0.2, SAP D 0.4, and SAP D 0.6) indicated that both the initial and final set times were overall delayed as the SAP addition increased, although SAP D 0.6 showed a slightly shorter initial set time than SAP D 0.4. This is because the more the SAP is added, the more the weaker spots are formed in the mortar matrix due to the existence of hydrogel (swollen SAP) with a much lower stiffness than the hydration products [37,50].

### 4.2. Effects of SAP Type and Content on the Strengths

**Figure 11:**
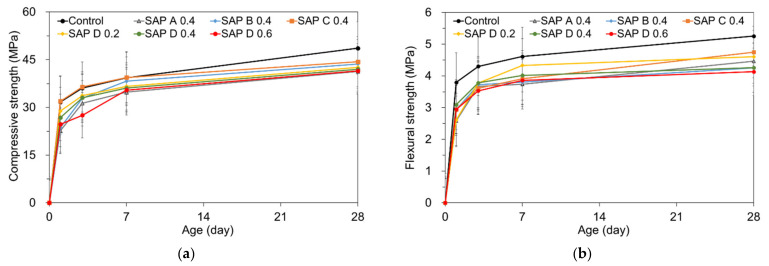
Effect of SAP addition on strengths: (**a**) compressive strength; (**b**) flexural strength.

The results of compressive strength and flexural strength tests are shown in Figure 11a,b. Both the compressive and flexural strengths overall decreased as the SAP addition increased, which was in good agreement with the findings of former investigations [22,25,26,41]. Again, this trend seems to be related with (1) the existence of the weaker spots filled with swollen hydrogel which are less capable of bearing stresses than the cement hydration products; and (2) increased macro-porosity resulting from the release of IC water during hydration of cement [39]. In addition, the strength reductions with an increased SAP addition could be attributed to the presence of angular pores formed by the irregularly pulverized SAPs as they can act as stress inducers [25,37].

**Figure 12:**
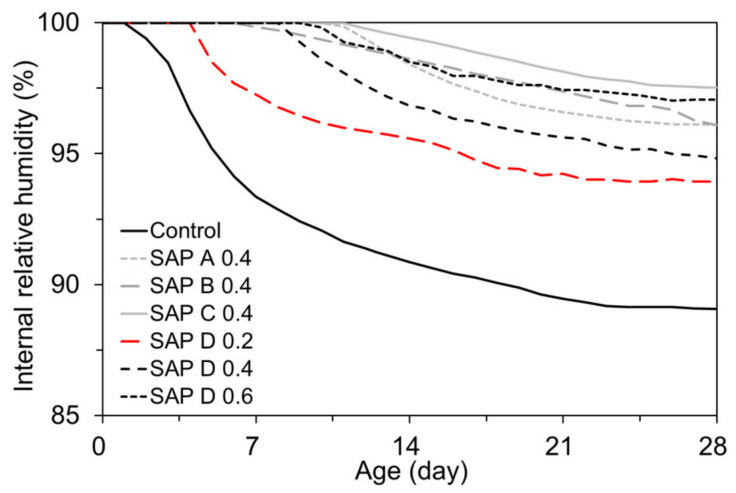
Effects of SAP addition on IRH changes.

While the compressive and flexural strengths at early ages were meaningfully lower than those of the control mixture particularly when more than 0.4% SAP was added, the fractional differences of strength with the control mixture were gradually reduced with aging, possibly due to the IC effect via SAP [49]. Note that the mixtures with SAP B and SAP C attained comparable or slightly higher compressive strengths than those with SAP A and SAP D at later ages, which is possibly due to the higher gel strength and superior moisture retention capacity of SAP C (as will be discussed in Figure 12) and the smaller pore size distribution created by SAP B. The strength reductions were more pronounced for the mixtures containing highly absorptive SAPs (SAP A and SAP D), similarly with the results of a previous study [51]. Even though the similar overall tendency in strength gaining over time and strength reductions with additions of SAP were found between the compressive strength and flexural strength, there was more inconsistency in the flexural strength than compressive strength. This is because the flexural strength reportedly exhibits larger variability than compressive strength as it is extremely sensitive to a number of factors such as specimen preparation, handling, and curing procedure [52]. The relatively lower compressive strength was obtained compared to the typical mixtures with a w/c of 0.3. This finding appears to be attributed to the high a/c relative to its low w/c [53] and the absence of mineral admixtures.

### 4.3. Effects of SAP Type and Content on the Autogenous Shrinkage and Internal Relative Humidity Changes

Figure 12 presents the IRH changes in each mixture measured for 28 days after final set. The IRH behavior remarkably differed depending on the type and content of SAP. First, when no SAP was added, the IRH drop started to take place soon after final set, whereas the initiation of IRH drop was considerably delayed when SAP were used. The IRH behaviors observed in the SAP D series (i.e., SAP D 0.2, SAP D 0.4, and SAP D 0.6) clearly revealed that as the SAP content increased, the initiation of IRH drop was meaningfully delayed: 4 days for SAP D 0.2, 7.9 days for SAP D 0.4, and 9.8 days for SAP D 0.6. This is because as more SAP was added with extra water, more curing water can be internally supplied via both osmotic pressure and IRH gradient between the matrix and SAP particles [54], which delayed the initiation of a self-desiccation process. This effect eventually reduced the 28-day IRH loss by 26.9% for SAP D 0.2, 47.3% for SAP D 0.4, and 55.4% for SAP D 0.6 compared to the control mixture. This trend was consistent with the results of previous studies [37,54,55]. It can also be noted from the 0.4% series (i.e., SAP A 0.4, SAP B 0.4, SAP C 0.4, and SAP D 0.4) that the rate and magnitude of IRH drop were affected by the cross-linking density and dimension of SAP. SAP C, which has the largest number of cross-links and the largest grain size, showed the most effective moisture retention performance. This is because the denser cross-links between polymer chains generate stronger elastic retraction, which in turn quickly releases the water absorbed in the SAP, raising the IRH [39]. On the other hand, SAP D 0.4 underwent relatively early and the most severe drying, probably because its high absorptivity (10.99 g/g) and medium-intensity cross-links prolong the moisture-holding time, which contributed to the slow release of the entrained water [41]. It is also interesting to note that SAP B, which has the smallest grain size, lost the IRH in a nearly constant rate. This was similar to its desorption kinetics assessed in a former study [41], and it seems to be mainly associated with the weaker van der Waals forces acting in SAP B. Additionally, the relatively faster loss of the absorbed water compared to the other types of SAP is likely related with the larger specific surface area and smaller nucleus of SAP B [56].

**Figure 13:**
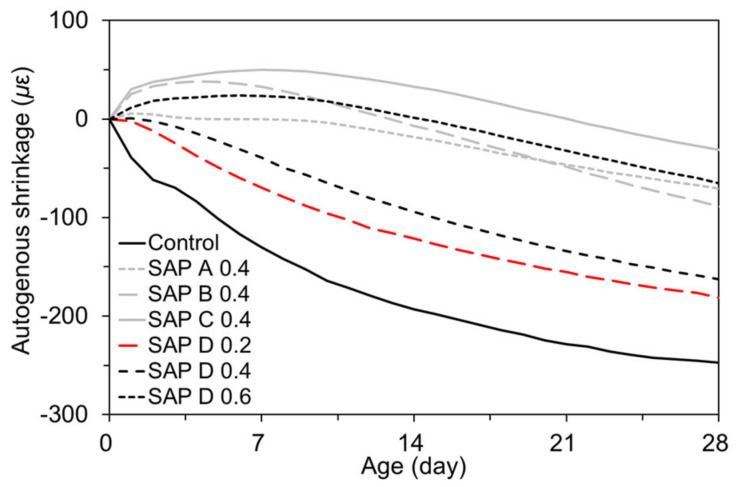
Effects of SAP addition on autogenous shrinkage development.

Figure 13 shows the developments of autogenous shrinkage in response to the IRH changes presented in Figure 12. Similar to the findings of previous studies [57,58], the trend of the autogenous shrinkage development was in good agreement with that of the IRH changes. This is because the IRH changes build up a pressure in capillary pores, and in turn drive the matrix to undergo shrinkage/swelling [59]. Overall, the mixtures with SAP experienced about 26.7% to 87.4% less autogenous shrinkage due to the IC effect provided by SAP with extra water. Some mixtures (i.e., SAP C 0.4, SAP B 0.4, and SAP D 0.6) even showed early swelling of approximately up to 50 µɛ, offsetting the subsequent autogenous shrinkage. SAP B 0.4 underwent the highest rate of autogenous shrinkage development because SAP B formed a finer pore structure in the matrix, thereby resulting in a stronger negative capillary pressure upon drying [60]. From the above findings, it was apparent that, under a “sealed” condition, the addition of SAP effectively counteracted the initiation of self-desiccation and corresponding autogenous shrinkage, and thus can be a promising measure to ensure effective IC of cement-based materials, particularly with a low w/b.

### 4.4. Effects of SAP Type and Content on the Restrained Stress Development

#### 4.4.1. Restrained Shrinkage Stress Development Due to Autogenous Shrinkage Only

**Figure 14:**
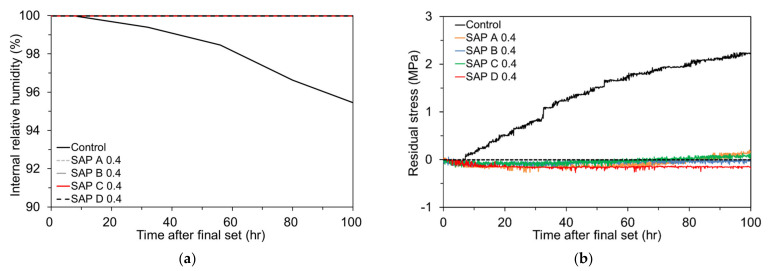
Effect of SAP type on: (**a**) IRH changes; and (**b**) restrained stress development.

Figure 14a shows the IRH changes in the mixtures reconstructed on the basis of Figure 12, highlighting the influences of SAP addition and type. Moreover, Figure 14b displays how the SAP addition and type affect the residual stress development in the ring under a sealed and isothermal condition for 100 h after final set. The residual stress was calculated based on Equation (1). It is noteworthy that the tendency of IRH changes observed in Figure 14a was quite similar to that of restrained stress development in Figure 14b. Whereas the control mixture with no SAP and extra curing water exhibited a sharp build-up of tensile stress as it experienced a rapid IRH drop soon after final set, no or very little increase in restrained stress was observed for the other mixtures with 0.4% SAP (i.e., SAP A 0.4, SAP B 0.4, SAP C 0.4, and SAP D 0.4). It is highly probable that the extra water supplied by SAP maintained the IRH of the mixtures very high (i.e., almost 100% RH) throughout the test period, and thus the autogenous shrinkage in those mixtures was mostly eliminated. These findings, in combination with the data shown in Figure 12 and Figure 13, clearly indicate that the early-age stress build-up and resulting cracking risk caused by autogenous shrinkage can be effectively mitigated by adding SAP and extra curing water.

**Figure 15:**
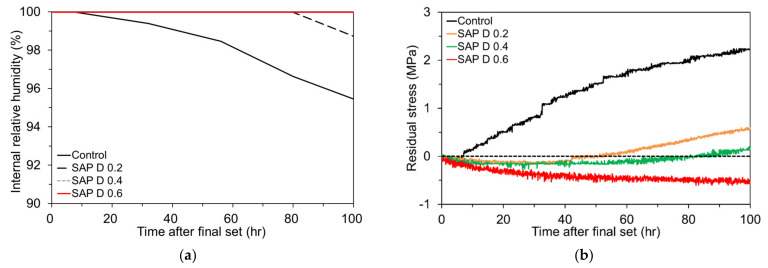
Effect of SAP content on: (**a**) IRH changes; and (**b**) restrained stress development.

The effect of SAP content on the IRH changes and corresponding early-age shrinkage stress development in the mortar ring is depicted in Figure 15a,b. Figure 15a was redrawn from Figure 12 for clarification. It is seen that the IRH of SAP D 0.2 commenced to diminish about 80 h after final set while that of SAP D 0.4 and SAP D 0.6 was maintained nearly 100% over the entire test period. It can be attributed to the fact that, as aforementioned, the lesser the SAP content, the lesser the IC water is stored in SAP and thus emptied earlier. Figure 15b shows the restrained stress development in the mortar ring when the IRH changes presented in Figure 15a were imposed. First, note that the residual tensile stress build-up was significantly lowered by adding SAP. Whereas the control mixture showed a residual tensile stress of 2.23 MPa after 100 h of final set, the other mixtures with SAP hardly allowed the accumulation of tensile stress. Particularly, no tensile stress was even developed for SAP D 0.6, which is benefited from the early expansion as confirmed in Figure 13. Furthermore, it can be seen the residual tensile stress was reduced almost in proportion to the SAP content. The residual stress after 100 h of final set was found to be 0.54 MPa for SAP D 0.2 (76% lower than control), 0.14 MPa for SAP D 0.4 (94% lower than control), and -0.56 MPa (compression) for SAP D 0.6. The discrepancies seem to be caused by the amount of extra curing water stored in SAP. Based on this finding, it is recommended to use more than 0.4% SAP (by mass of cement) in order to adequately limit the autogenous shrinkage development at early ages although dosage adjustment may be needed depending on the absorptivity of SAP and mixture proportions. Lastly, it is also of great importance to see that the IRH changes in Figure 15a were well matched with the restrained stress development in Figure 15b. While no or negligible residual tensile stress was developed for SAP D 0.4 and SAP D 0.6, of which the RH was maintained nearly 100% for the entire test period, SAP D 0.2 began to show a tensile stress build-up after about 60 h, as some IRH loss occurred as noted in Figure 15a. As discussed earlier, the control mixture initiated a gradual IRH drop shortly after final set, which in turn caused a steady increase in tensile stress over the test period.

#### 4.4.2. Restrained Shrinkage Cracking Due to Autogenous Shrinkage and Delayed External Drying

**Figure 16:**
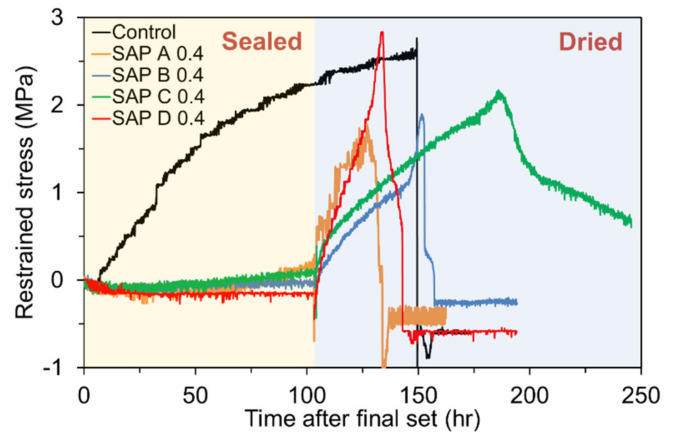
Restrained stress development in mortar ring specimens with different SAP types when drying initiated after 100 h of final set.

**Figure 17:**
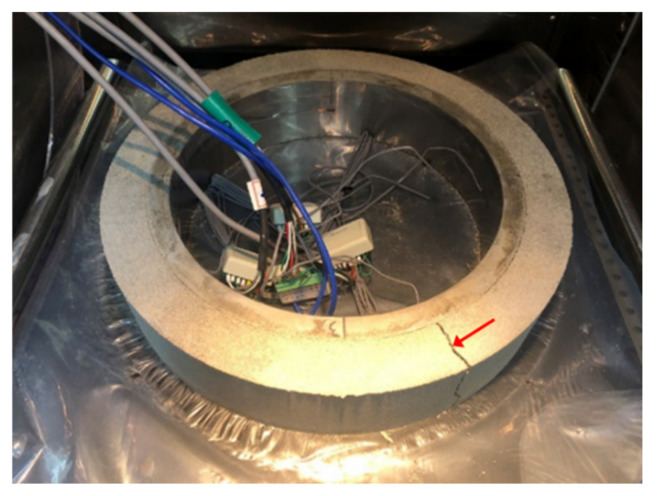
Cracking of mortar ring specimen due to restrained shrinkage.

To investigate the shrinkage cracking sensitivity to different SAP types and contents, the ring specimens were unsealed after 100 h of final set, except the control mixture; the control mixture was continuously kept in a sealed condition until it cracked. The residual stress developments in the control specimen and the specimens with different SAP types are compared in Figure 16. As mentioned previously, the control mixture began to experience a steady tensile stress build-up shortly after final set due to severe self-desiccation, which finally cracked in 150 h without external drying. Note that the tensile stress was instantaneously relieved upon failure. Figure 17 shows the cracked specimen upon completion of the testing. The results also indicate that the specimens with different SAP types exhibited a different magnitude and rate of stress development after drying. For SAP C 0.4 which had the latest onset of self-desiccation, highest early autogenous expansion, and relatively lower tensile stiffness (estimated based on the measured flexural strength in Figure 11b), the rate of tensile stress development was noticeably low, and thus cracked the latest (about 187 h). Similarly, SAP B 0.4 which had the second highest early autogenous expansion next to SAP C 0.4 and comparably lower tensile stiffness also resulted in the lowest rate of tensile stress development. The earlier cracking of SAP B 0.4 than SAP C 0.4 was observed, which is attributed to its lower strength than SAP C 0.4. On the contrary, SAP D 0.4 had the greatest IRH loss, autogenous shrinkage, and tensile stiffness, which yielded the highest magnitude and rate of stress development. SAP A 0.4 which had the second greatest autogenous shrinkage exhibited a comparable rate of tensile stress development to SAP D 0.4 but cracked earlier than SAP D 0.4 because of its lower strength. Basically, there was a rough tendency that the onset of shrinkage cracking was delayed as the cross-linking density of SAP increased as it determines the time of water release. Consequently, such discrepancies in the rate and magnitude of stress development, and cracking behavior among the mixtures seem to arise from the combined effect of age-dependent changes in autogenous shrinkage, tensile strength, stiffness, creep, and fracture properties. It can also be seen in Figure 16 that there was a notable difference in post-cracking behavior between the control specimen and the specimens containing SAP. While the control specimen showed a brittle behavior at cracking, the specimens with SAP showed a slightly ductile post-cracking behavior, which appears to be attributed to the high ductility of the hydrogel dispersed in the matrix. It should be also mentioned that the specimens cracked at the stress levels lower than the flexural strengths presented in Figure 11b primarily because they failed in a tensile mode, which is more critical than a flexural failure mode [61]. Plus, while the strength specimens were consistently cured in 95% RH until the specified ages are reached, the ring specimens were exposed to the ambient after 100 h of final set for faster drying, which led to a lack of curing water required for cement hydration and surface micro-damage due to moisture shock.

**Figure 18:**
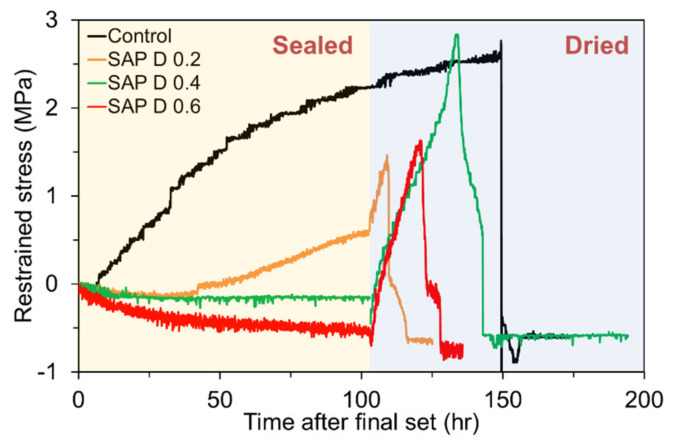
Restrained stress development in mortar ring specimens with different SAP contents when drying initiated after 100 h of final set.

Figure 18 depicts the influence of SAP content on the restrained stress development in the mortar ring. The trend displayed by the results was quite similar to the findings of a previous study, wherein prewetted lightweight aggregate was used for IC [62]. Again, when no SAP was added, there was a rapid accumulation of tensile stress throughout the test period due to severe autogenous shrinkage, and thus the specimen eventually cracked without external drying. On the other hand, the other three mixtures with SAP showed much lower residual stress increments when sealed. However, the specimens with SAP started to experience a rapid increase in tensile stress when drying began at 100 h. As a result, the specimens finally cracked in 10 to 34 h of external drying. In the external drying phase, there were no substantial differences in the rate of tensile stress development among the SAP D 0.2, SAP D 0.4, and SAP D 0.6 mixtures, supporting that the addition of SAP (regardless of its content) was rarely effective for the control of drying shrinkage cracking potential in cement-based mixtures. There was no strong relevance between the SAP content and time of cracking, rate of restrained stress development, and peak stress at failure because the SAP addition affected the complex evolutions of various age-dependent material properties such as strength, elastic modulus, fracture energy absorption, and stress relaxation, as well as moisture-induced material behavior. Above all, the most meaningful finding in this part of the study is that while the addition of SAP apparently provided a huge benefit to limit the tensile stress build-up caused by internal drying (autogenous shrinkage), it was hardly effective when subjected to external drying (drying shrinkage). Further detailed research is needed to better understand and verify the obtained results.

#### 4.4.3. Restrained Stress Development Due to Immediate External Drying

**Figure 19:**
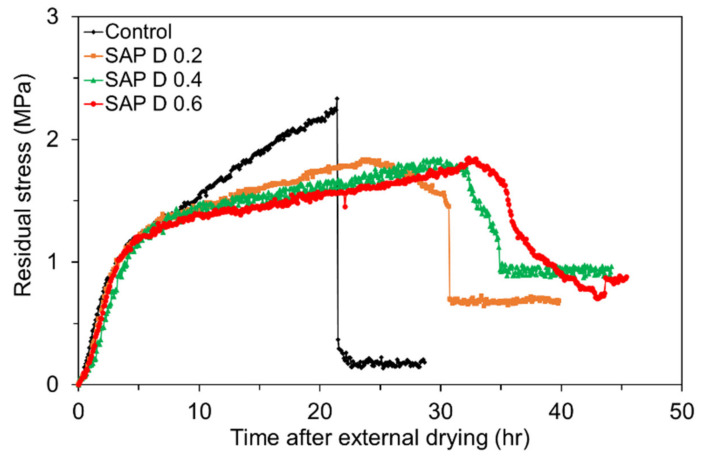
Restrained stress development in mortar ring specimens with different SAP contents when external drying initiated immediately final set.

The residual stress developments in the mixtures with various SAP contents are compared in Figure 19. Both the control mixture and mixtures with SAP were exposed to the controlled ambient as soon as the final set was reached (about 24 h after mixing). First, note that the rate of residual stress development was lower for the SAP mixtures than the control mixture due to the IC effect. As the SAP content increased, the rate of residual stress development tended to be slightly reduced. The peak residual tensile stress in the SAP mixtures was about 21.5% lower than that of the control mixture on average, which is attributed to the reduced early-age strength with additions of SAP as revealed in Figure 12. It is also interesting to see that the mixtures with SAP cracked later than the control mixture, and the time of cracking monotonically increased with an increase in SAP content; the results differed compared to what observed in Figure 18 because, as discussed earlier, the initial curing via SAP (for 100 h) notably changed the hydration kinetics, microstructure development, and age-dependent material properties and behavior. The time of cracking was 21.4 h for the control mixture, 30.7 h for SAP D 0.2, 31.9 h for SAP D 0.4, and 33.1 h for SAP D 0.6 as the IC water supply via SAP delayed the rate of internal drying. The results were in good agreement with those in former studies [23,48]. Yet, it is of utmost importance to identify that the addition of SAP was not sufficient to reduce the drying shrinkage cracking potential at early ages, while it was quite effective in alleviating the cracking potential due to autogenous shrinkage. Lastly, as with the results shown in Figure 16, it was obvious that some ductile behavior occurred in the SAP mixtures at failure, and it became more pronounced as the SAP content increased. This finding proves that the mortar became more compliant with the addition of SAP.

## 5. Conclusions

A series of experimental characterizations was conducted to assess how differently mortars incorporating different types and contents of superabsorbent polymer (SAP) behave under autogenous and drying conditions. Internal relative humidity (IRH), autogenous shrinkage, restrained shrinkage stress development, and cracking potential were measured for the evaluations. Furthermore, the initial and final set times and strengths were measured for systemic analysis of the obtained results. The key findings of this study can be drawn as follows:

- The greater the cross-linking density (i.e., gel strength), the shorter the set times. Both the initial and final set times were overall delayed with an increased SAP content.
- The additions of SAP had an adverse impact to the strengths as both the compressive and flexural strengths were overall reduced as the SAP content increased. The particle size and cross-linking density of SAP also affected the strength characteristics as they determine the time of water release from SAP and pore structure of the matrix.
- The proposed test methods were proven to be valid as the measured shrinkage and residual stress profiles correlated well with the measured IRH changes.
- The addition of SAP up to 0.6% (by mass of cement) successfully reduced the IRH loss by up to 87.4% compared to the control mixture. There was an obvious trend that as the SAP content increased, both the rate and magnitude of IRH drop decreased due to the internal curing (IC) effect. The rate of IRH changes was also partly dependent on the cross-linking density and particle size of SAP.
- As expected, the IC via SAP effectively limited the residual stress build-up due to autogenous shrinkage. In particular, no or negligible tensile residual stresses occurred due to self-desiccation when more than 0.4% SAP was added. This finding indicates that adding SAP with an appropriate amount of extra curing water can be a promising strategy to mitigate the autogenous shrinkage-induced cracking potential and to extend the service life of high-performance concrete.
- However, the addition of SAP, irrespective of its content, hardly contributed to the gaining of sufficient resistance to cracking when directly exposed to drying environment because a rapid increase in residual stress and eventual cracking were observed as soon as the specimen was unsealed for external drying.

## Figures and Tables

**Table 1:** Chemical composition and physical properties of cement.

Chemical Composition (%)								Fineness (m^2^/kg)	Specific Gravity (-)
SiO_2_	Al_2_O_3_	Fe_2_O_3_	CaO	MgO	SO_3_	K_2_O	Na_2_O		
19.7	5.33	2.90	61.5	3.81	2.54	0.86	0.18	370	3.15

**Table 2:** General properties of superabsorbent polymer (SAPs) used.

SAP ID	Dry Particle Size	Cross-Linking Density ^1^	Rate of Moisture Uptake ^1^	Capacity of Moisture Uptake
SAP A	#80–20 mesh (177–841 μm)	Low	Low	High (12.70 g/g)
SAP B	#120–80 mesh (125–177 μm)	Medium	High	Medium (8.75 g/g)
SAP C	#80–20 mesh (177–841 μm)	High	Low	Low (4.82 g/g)
SAP D	#100–40 mesh (149–400 μm)	Medium	Medium	High (10.99 g/g)

^1^ Relative scale.

**Table 3:** Chemical and physical properties of SAPs used.

Chemical Nomenclature	Chemical Formula	Molar Mass (g/mol)	Constitutional Formula	Density (g/cm^3^)
Poly(sodium prop-2-enoate)	(C3H3NaO2)n	Variable	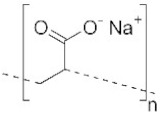	1.22

**Table 4:** Mixture proportions of mortar.

Mixture ID	Effective w/c	Weight per Unit Volume (kg/m^3^)					
		Cement	Fine Aggregate	Water	SAP	IC Water	Water Reducer
**Control**	0.3	604.2	1661.6	181.3	-	-	3.02
SAP A 0.4		586.2	1612.1	175.9	2.35	29.8	2.93
SAP B 0.4		591.7	1627.2	177.5	2.37	20.7	2.96
SAP C 0.4		597.3	1642.6	179.2	2.39	11.5	2.99
SAP D 0.2		596.3	1639.8	178.9	1.19	13.1	2.98
SAP D 0.4		588.6	1618.7	176.6	2.35	25.9	2.94
SAP D 0.6		581.1	1598.0	174.3	3.49	38.3	2.91

**Table 5:** Fitting parameters estimated for each mixture.

Mixture ID	*y* = *a* exp (*bx*)	
	*a*	*b*
Control	0.1111	1.1208
SAP A 0.4	0.4571	0.5062
SAP B 0.4	0.8539	0.4244
SAP C 0.4	0.3381	0.7278
SAP D 0.2	0.4571	0.5062
SAP D 0.4	0.4575	0.5344
SAP D 0.6	0.7776	0.3414

## Data Availability

The data presented in this study are available within the article.
